# Optimization of CO_2_/H_2_ Separation over Ba-SAPO-34 Zeolite Membrane Synthesized by Microwave Heating

**DOI:** 10.3390/membranes12090850

**Published:** 2022-08-30

**Authors:** Tiffany Yit Siew Ng, Vinosha Viriya, Thiam Leng Chew, Yin Fong Yeong, Abdul Latif Ahmad, Chii-Dong Ho, Zeinab Abbas Jawad

**Affiliations:** 1 CO2 Research Centre (CO2RES), Institute of Contaminant Management, Universiti Teknologi PETRONAS, Seri Iskandar 32610, Perak, Malaysia; 2 Department of Chemical Engineering, Universiti Teknologi PETRONAS, Seri Iskandar 32610, Perak, Malaysia; 3 School of Chemical Engineering, Universiti Sains Malaysia, Nibong Tebal 14300, Penang, Malaysia; 4 Department of Chemical and Materials Engineering, Tamkang University, New Taipei City 25137, Taiwan; 5 Department of Chemical Engineering, College of Engineering, Qatar University, Doha P.O. Box 2713, Qatar

**Keywords:** zeolite membrane, Ba-SAPO-34, CO_2_/H_2_ separation, response surface methodology

## Abstract

CO_2_/H_2_ separation using membrane technology is an important research area in order to obtain high purity hydrogen as one source of clean energy. Finding a suitable inorganic membrane is one of the critical issues, which needs to be explored for CO_2_/H_2_ separation. In the present study, Ba-SAPO-34 zeolite membrane was synthesized and followed by a modification process. CO_2_/H_2_ separation of the membrane was investigated by varying the independent process variables (CO_2_ % in the feed, pressure difference across the membrane and temperature). Modeling and optimization for the responses (CO_2_/H_2_ separation selectivity and CO_2_ permeance) was performed by applying response surface methodology and central composite design, which is available in Design Expert software. The accuracy of the models in predicting the response was tested by comparing with the experimental value of response and the two values were in good agreement. The optimization of the models gave CO_2_ permeance of 19.23 × 10^−7^ mol/m^2^ s Pa and CO_2_/H_2_ separation selectivity of 11.6 at 5% CO_2_ in the feed, a pressure difference of 100 kPa, and temperature of 30 °C for Ba-SAPO-34 zeolite membrane.

## 1. Introduction

In recent years, gas separation has received enormous attention among researchers due to the issues of energy security and global climate change. Numbers of articles for gas separation processes have been published [1,2,3,4,5]. Hydrogen (H_2_) separation technologies have gained increasing importance nowadays since hydrogen is one of the important chemical sources for industries. It is also one the main energy sources for transportation fuel and electrical power generation [6]. Separation and purification are important technologies in processes for H_2_ production, such as thermochemical processes. In order to obtain high purity H_2_, separation of H_2_ from carbon dioxide (CO_2_) is one such important area [5].

Common separation techniques for H_2_ separation from CO_2_ are physical absorption with solvents, pressure swing adsorption and cryogenic distillation [5,7]. However, these processes have a number of drawbacks such as complexity of the system, high energy consumption for solvent regeneration, equipment corrosion and flow problems caused by viscosity of solvent [8,9]. Membrane-based technology appears to be a potential alternative for H_2_ separation in view of its advantages such as sustainable operation and relatively low energy consumption [7]. Palladium membranes have been extensively studied for H_2_ separation due to its high hydrogen selectivity [10,11,12]. However, the usage of palladium and its alloys have a number of disadvantages, including a high sensitivity to chemicals (i.e., sulphur, chlorine and carbon monoxide in most applications) and their extremely high cost [13]. Polymeric membrane are other candidates for separation of H_2_ in view of their low cost and low energy requirement [14,15]. However, the application of polymeric membrane in H_2_ separation is limited by the disadvantages such as the low mechanical stability of rubbery polymers [16].

Zeolite membranes are microporous inorganic membranes that are gaining increasing interests for CO_2_/H_2_ separation. Zeolite membranes possess advantages such as uniform pore structure and high chemical stability [17,18]. Different types of zeolite membranes have been studied for gas permeation and separation. These include CHA [17], FAU-type [19,20], PWN-type [21], A-type [22,23,24,25], MFI-type [26,27,28,29,30,31], DDR [32,33], T-type [34,35] and silicoaluminophosphate (SAPO) membranes [18,36,37]. There have been a number of studies on SAPO-34 zeolite membrane for gas permeation and separation due to its small pore structure [18,36,37,38,39,40,41,42,43,44,45,46]. Owing to the pore size of SAPO-34 which is close to the kinetic diameter of the CO_2_ molecule, the SAPO-34 zeolite membrane has a high potential for separation of H_2_ from CO_2_. Hong et al. [39] reported that the SAPO-34 membrane selectively separated CO_2_ from the CO_2_/H_2_ binary gas mixture at low temperature and the membrane became H_2_ selective at a high temperature of 200 °C.

Design of Experiments is commonly used to perform optimization for the process parameters [47]. Response surface methodology (RSM), available in Design of Experiments, is a statistical tool that could allow reduction in the required numbers of experiments and could be used to investigate the effect of the significant process variable and the effect of the variables’ interaction on the process [48]. There have been numerous studies that have applied RSM and hence showed RSM as an effective tool for optimization of the process [49,50,51,52].

Our previous work [53] has shown that CO_2_/CH_4_ separation was selectivity improved from 30 for the H-SAPO-34 zeolite membrane to 103 for the Ba-SAPO-34 zeolite membrane. In the present work, Ba-SAPO-34 zeolite membrane was formed by modifying the pre-synthesized H^+^-form of SAPO-34 (H-SAPO-34) zeolite membrane. The Ba-SAPO-34 zeolite membrane was subjected to the CO_2_/H_2_ separation process by varying three process variables, which are CO_2_ % (concentration) in the feed, pressure difference and temperature. The objective of the current study was to perform optimization on the operating process conditions of the membrane separation for the CO_2_/H_2_ separation selectivity and CO_2_ permeance. In current work, CO_2_ permeance was reported instead of H_2_ permeance because the Ba-SAPO-34 membrane was found to be CO_2_-selective over the ranges of the process variables studied.

## 2. Materials and Methods

### 2.1. Preparation of Zeolite Membrane

H-SAPO-34 membrane was deposited on α-alumina disc and then followed by a modification to the Ba-SAPO-34 membrane by following the procedures described in our previous work [43,53]. The synthesis precursor with the molar composition of Al_2_O_3_:P_2_O_5_:1.2TEAOH:0.3SiO_2_:80H_2_O was prepared by mixing deionized water, aluminium isopropoxide (Al(i-C_3_H_7_O)_3_, 98%, Merck, Darmstadt, Germany), tetraethylammonium hydroxide (TEAOH, 35 wt%, Sigma–Aldrich, St. Louis, MI, USA), phosphoric acid (H_3_PO_4_, 85%, Sigma–Aldrich) and Ludox AS-40 colloidal silica sol (40 wt%). The synthesis precursor was poured into a Teflon-lined vessel with α-Alumina disc placed in the vessel. The filled Teflon-lined vessel was heated at 200 °C for 2 h in a microwave oven (MARS 5, CEM Corporation, Matthews, NC, Canada). When the microwave heating was done, rinsing and drying were performed on the membrane. The procedures for heating, rinsing and drying were repeated three times. Calcination was performed on the H-SAPO-34 membrane at 400 °C for 15 h in a furnace. In order to modify the H-SAPO-34 membrane to the Ba-SAPO-34 membrane, ion-exchange was performed on the H-SAPO-34 membrane at 70 °C for 5 h by using ion-exchange solution containing Ba^2+^. Rinsing the membrane with ethanol and followed by drying the membrane at 100 °C overnight were then carried out. The characterization works of the Ba-SAPO-34 membrane were described in our previous work [43,53].

### 2.2. Design of Experiments

By using Design Expert software version 6.0.6 (STAT-EASE Inc., Minneapolis, MN, USA), Design of Experiments was applied for investigating the CO_2_/H_2_ separation. The modeling and analysis of problems, which include the generation of model equations by using experimental data, determination of the effect of variables and variables’ interaction on the responses and optimization studies on the responses, were performed by using RSM coupled with central composite design (CCD) [54,55].

Three independent variables were studied for CO_2_/H_2_ separation in the current study, which include CO_2_ % in the feed, pressure difference and temperature, as shown in Table 1. The factor code for CO_2_ % in the feed, pressure difference and temperature is C, B and A, respectively. As shown in Table 1, the low level and high level are represented by −1 and +1, respectively. CO_2_ separation selectivity and CO_2_ permeance are the responses that were investigated in the current study.

Equations (1) and (2) show the polynomial that can be investigated by using Design Expert software for the approximation for the relationship between response (*y*) and the set of independent variables [50,54,56]:

First order model:

(1) $$y=\beta_{0}+\beta_{1}x_{1}+\beta_{2}x_{2}\_+\ldots +\beta_{n}x_{n}+\varepsilon$$

Second order model:

(2) $$y=\beta_{0}+\sum_{i=1}^{n}\beta_{i}x_{i}+\sum_{i=1}^{n}\beta_{ii}x_{i}^{2}+\sum \sum_{i<j}^{}\beta_{ij}x_{i}x_{j}+\varepsilon$$

where *y* is the response, $x_{i}$ and $x_{j}$ are the independent variables, $x_{i}x_{j}$ is the first order interaction between $x_{i}$ and $x_{j}$, $\beta_{0}$, $\beta_{i}$, $\beta_{ii}$ and $\beta_{ij}$ is the regression coefficient for intercept, linear, quadratic and interaction terms, respectively, n is the number of independent variables and ε is the error. 

### 2.3. CO_2_/H_2_ Gas Separation Studies

The Ba-SAPO-34 membrane was sealed in a stainless steel module using silicone gaskets and was subjected to CO_2_/H_2_ separation studies. Mass flow controllers were used to feed CO_2_ and H_2_ gases to the membrane module The CO_2_ concentration in the feed was varied. The permeate pressure was kept at atmospheric pressure. Back pressure regulator was used to adjust the feed pressure so that the pressure difference across Ba-SAPO-34 membrane can be varied. The temperature for gas separation was varied by changing the temperature of an electronic-controlled oven where the membrane module was located. Online gas chromatography (PERKIN ELMER, CLARUS 500) equipped with CARBOXEN-1010 column and thermal conductivity detector, was used to analyze the composition of the permeate and retentate exit streams.

Permeance, $P_{i}$ (mol/m^2^ s Pa) of the gas was determined by using Equation (3).

(3) $$P_{i}=\frac{J_{i}}{\Delta p_{i}}$$

where $\Delta p_{i}$ is the partial pressure difference of gas *i* across the membrane (Pa), $J_{i}$ is the flux of gas *i* (mol/m^2^ s), the gas *i* may corresponds to CO_2_ or H_2_.

The CO_2_/H_2_ separation selectivity, $\alpha_{CO_{2}/H_{2}}^{}$ was determined by using Equation (4).

(4) $$\alpha_{CO_{2}/H_{2}}^{}=\frac{P_{CO_{2}}}{P_{H_{2}}}$$

## 3. Results

### 3.1. Characterization Results of Ba-SAPO-34

The Ba-SAPO-34 membrane was characterized by using Scanning Electron Microscopy (SEM) and High-Resolution Transmission Electron Microscopy (HRTEM) in our previous work [43]. The top view SEM image and cross-sectional view SEM image of Ba-SAPO-34 membrane can be found in our previously published works [43]. It was observed from the top view SEM image of the Ba-SAPO-34 membrane that the membrane consists of orthorhombic zeolite crystals with a size of approximately 1 μm [43]. Meanwhile, the cross-sectional view SEM image of the Ba-SAPO-34 membrane showed that Ba-SAPO-34 membrane layer thickness is 4 μm approximately [43]. On the other hand, the HRTEM image of Ba-SAPO-34 can also be found in our previously published works [43] and the HRTEM showed Ba-SAPO-34 with pore channel diameter of less than 0.5 nm.

### 3.2. Experiment Design Matrix

In the current study, a total of 20 experiment runs for sets of independent variables (C: CO_2_ % in the feed, B: pressure difference and A: temperature) was suggested by CCD for the CO_2_/H_2_ gas separation studies as shown in Table 2. The values of CO_2_/H_2_ separation selectivity and CO_2_ permeance, which were obtained from experimental work, are shown in Table 2 as well. The CO_2_ permeance was in the range of 1.96 to 19.23 × 10^−7^ mol/m^2^ s Pa and the CO_2_/H_2_ separation selectivity was in the range of 2.3 to 12.2. The experimental runs at the temperature of 105 °C, pressure difference of 300 kPa and 27.5% CO_2_ in the feed were repeated another five times (run 15–20 as shown in Table 2) in order to check for the reproducibility of the data. The low values of standard deviations (0.01 for CO_2_ permeance and 0.05 for CO_2_/H_2_ separation selectivity) for the repeated runs indicates good reproducibility of the responses.

### 3.3. Response Surface Modeling

“Inverse” transformation was used to analyze the responses of CO_2_ permeance and CO_2_/H_2_ separation selectivity as defined in Equation (5).

(5) $$y\prime =\frac{1}{y}$$

where *y* is the value of the response and *y’* is the transformed value. “Inverse” transformation was applied because this function was able to model and predict the experimental data very well. The CO_2_ permeance and CO_2_/H_2_ separation selectivity was modeled, analyzed in the form of 1/(CO_2_ permeance) and 1/(CO/H_2_ separation selectivity) respectively.

#### 3.3.1. Response Surface Modeling of CO_2_ Permeance

The Analysis of Variance (ANOVA) of the CO_2_ permeance is shown in Table 3. Equation (6) shows the chosen quadratic model to reflect the relationship between the independent variables and the response.

(6) $$\begin{array}{ll} 1/{(\mathrm{CO}}_{2}\;\mathrm{Permenace})\; & =+0.30+0.051A+0.037B+0.14C+0.017A^{2}-0.026B^{2}\\ & -0.018C^{2}-0.030\mathrm{AB}+0.026\mathrm{AC}+0.011\mathrm{BC} \end{array}$$

where C, B and A correspond to the coded value of CO_2_ % in the feed, pressure difference and temperature, respectively. 

The model F-value of 29,876.61 implies the model was significant. Temperature (A) gave the highest F value of 26,945.27, indicating that it had the most significant effect on CO_2_ permeance compared to pressure difference (B) and CO_2_ % in the feed (C).

In order to have the terms of the model to be significant at the 95% confidence level, the values of probability should be less than 0.0500 (“Prob > F” less than 0.0500). In this case, all the terms (A, B, C, A^2^, B^2^, C^2^, AB, AC and BC) were found to be significant for the model of 1/(CO_2_ permeance). The “Lack of Fit F-value” of 1.16 implied that the Lack of Fit was not significant relative to the pure error. It is good to have non-significant Lack of Fit.

**Figure 1:**
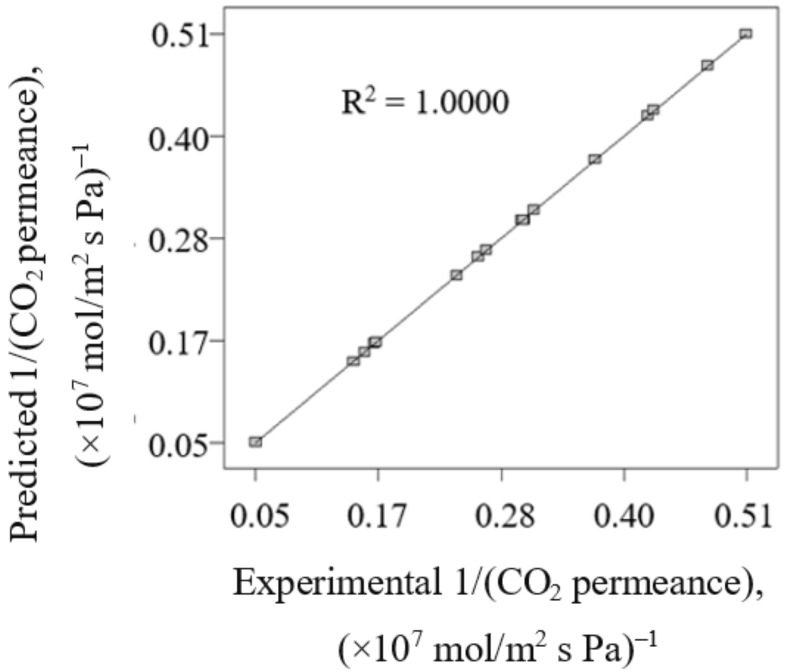
The comparison between predicted 1/(CO_2_ permeance) attained by using Equation (6) with the experimental 1/(CO_2_ permeance).

Figure 1 presents the comparison between predicted 1/(CO_2_ permeance) attained by using Equation (6) with the experimental 1/(CO_2_ permeance). Good agreement between predicted 1/(CO_2_ permeance) and experimental 1/(CO_2_ permeance) is indicated by the correlation coefficient value (R^2^) of 1.000. Hence, this reflects the high accuracy of the generated model Equation (6) to predict the 1/(CO_2_ permeance) in current work.

**Figure 2:**
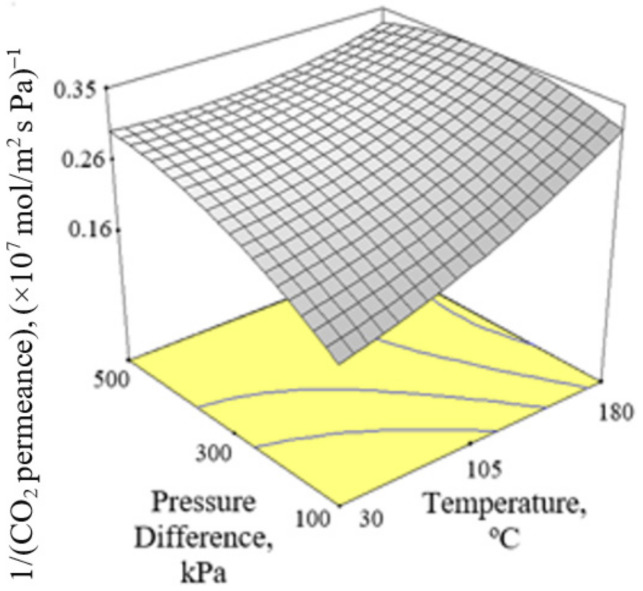
Effect of interaction between temperature and pressure difference on the 1/(CO_2_ permeance) at 27.5% CO_2_ in the feed.

**Figure 3:**
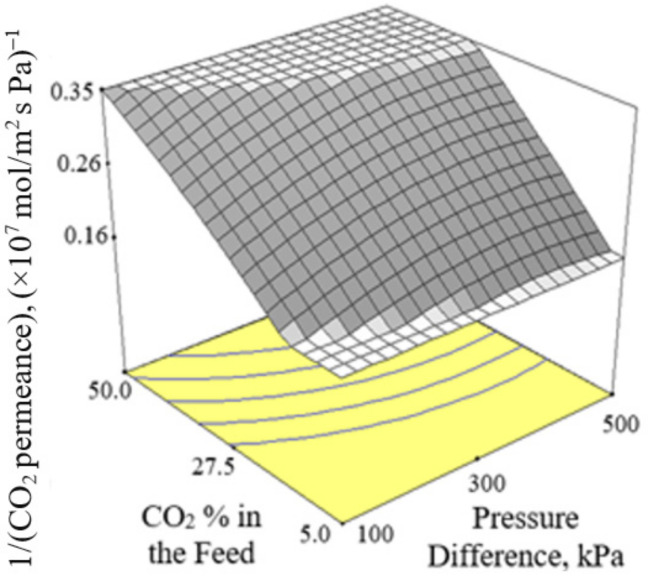
Effect of interaction between CO_2_ % in the feed and pressure difference on the 1/(CO_2_ permeance) at 105 °C.

**Figure 4:**
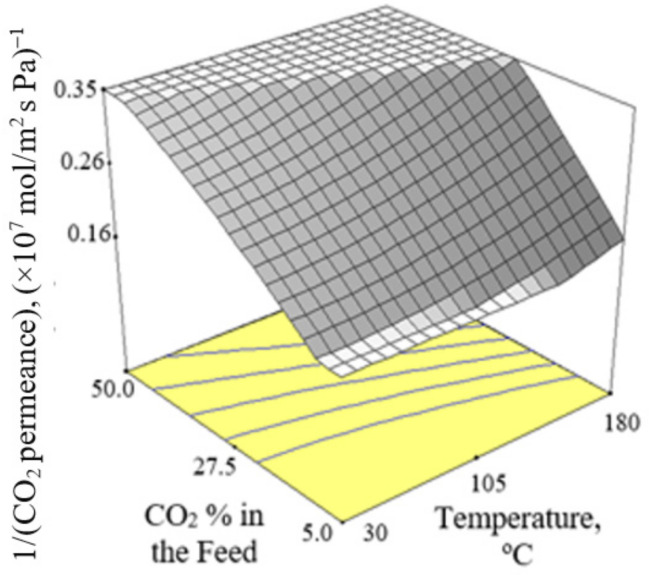
Effect of interaction between CO_2_ % in the feed and temperature on the 1/(CO_2_ permeance) at 300 kPa pressure difference.

Figure 2, Figure 3 and Figure 4 present the plots showing the effect of interaction between different independent variables on the 1/(CO_2_ permeance). It can be observed from Figure 2 that the 1/(CO_2_ permeance) increased with temperature for all three levels of pressure difference. A similar trend was reported by Li et al. [42]. When the separation temperature increased from 30 to 180 °C, the surface coverage declined and the CO_2_ diffusivity increased. The decline in surface coverage prevailed the increment in diffusivity when the temperature increased. Subsequently, this resulted in decline in CO_2_ permeance, and hence increment in the 1/(CO_2_ permeance) when the temperature increased. The 1/(CO_2_ permeance also increased with pressure difference as shown in Figure 2. In the current study, gas permeance was calculated by dividing the gas flux with partial pressure difference across the membrane. The increase in CO_2_ partial pressure gradient was more than the increase in CO_2_ surface coverage gradient, hence leading to a decrease in CO_2_ permeance with an increase in pressure difference of 100–500 kPa across the membrane in the current study. Figure 3 and Figure 4 show that the 1/(CO_2_ permeance) increased with an increase in CO_2_ % in the feed from 5 to 50%. When the CO_2_ % in the feed increased, the CO_2_ permeance decreased. The increase in the CO_2_ loading approached saturation in the membrane and thus resulted in drop in CO_2_ permeance as observed and reported by Hong et al. [39].

#### 3.3.2. Response Surface Modeling of CO_2_/H_2_ Separation Selectivity

The ANOVA of the CO_2_/H_2_ separation selectivity is shown in Table 4. Equation (7) shows the chosen quadratic model to reflect the relationship between the independent variables and the response.

(7) $$\begin{array}{ll} 1/{(\mathrm{CO}}_{2}{/H}_{2}\;\mathrm{Separation}\;\mathrm{Selectivity})\; & =+0.32+0.12A-0.005579B+0.060C-{0.020A}^{2}\\ & +0.010B^{2}-{0.039C}^{2}-0.046\mathrm{AB}+0.043\mathrm{AC}\\ & -0.015\mathrm{BC} \end{array}$$

where C, B and A correspond to the coded value of CO_2_ % in the feed, pressure difference and temperature, respectively.

The model was significant in view of its F-value of 391.84. The significance of variable’s effect on CO_2_/H_2_ separation selectivity decreased in the order of temperature (A) > CO_2_ % in the feed (C) > pressure difference (B) with the F-value in the order of 2209.94 > 586.84 > 5.03, respectively. It is shown in Table 4 that A, B, C, A^2^, C^2^, AB, AC, BC were significant terms for the model of 1/(CO_2_/H_2_ separation selectivity). However, the term of B^2^ was included in Equation (7) to obtain a hierarchy model. The “Lack of Fit F-value” of 4.39 implied that the Lack of Fit was not significant relative to the pure error due to noise.

**Figure 5:**
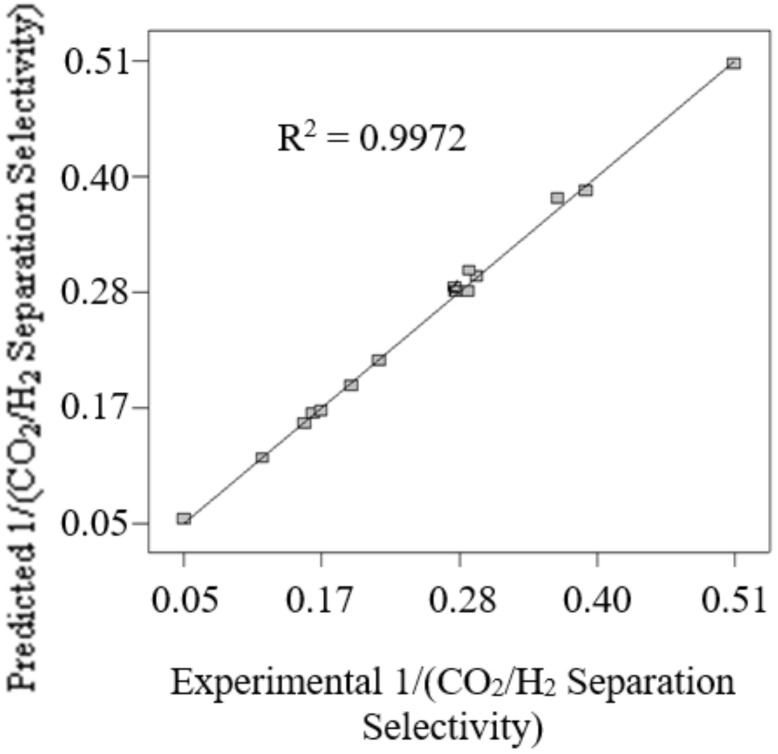
The comparison of the predicted 1/(CO_2_/H_2_ separation selectivity) attained by using Equation (7) with the experimental 1/(CO_2_/H_2_ separation selectivity).

Figure 5 presents the comparison of the predicted 1/(CO_2_/H_2_ separation selectivity) attained by using Equation (7) with the experimental 1/(CO_2_/H_2_ separation selectivity). Good agreement between predicted 1/(CO_2_/H_2_ separation selectivity) and experimental 1/(CO_2_/H_2_ separation selectivity) is indicated by the correlation coefficient value (R^2^) of 0.9972. Hence, this reflects the high accuracy of the generated model Equation (7) to predict the 1/(CO_2_/H_2_ separation selectivity) in the current work.

**Figure 6:**
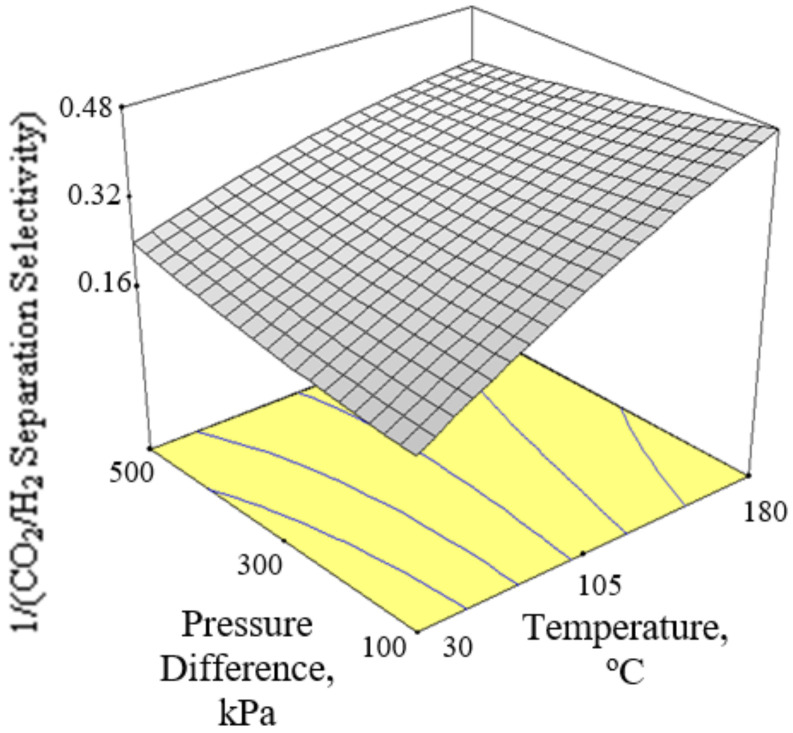
Effect of interaction between temperature and pressure difference on the 1/(CO_2_/H_2_ separation selectivity) at 27.5% CO_2_ in the feed.

**Figure 7:**
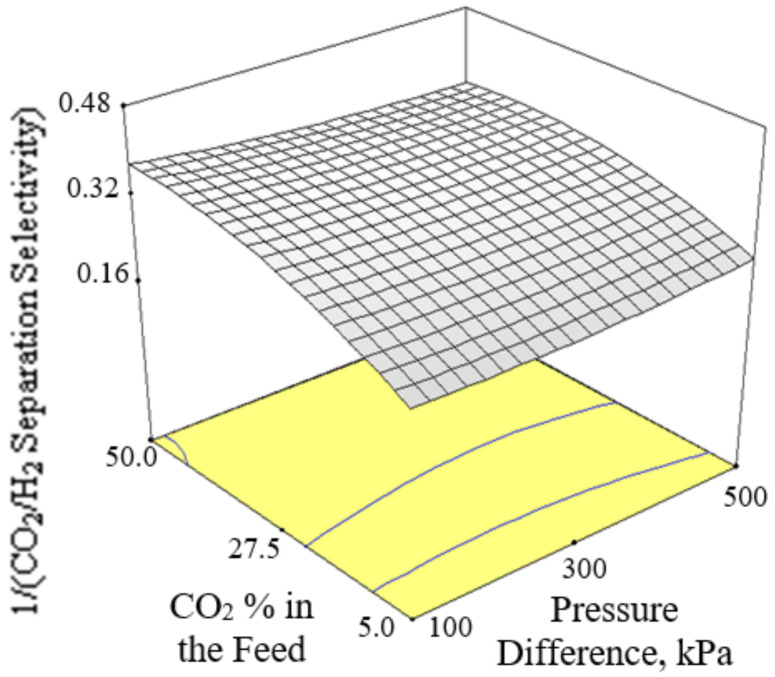
Effect of interaction between CO_2_ % in the feed and pressure difference on the 1/(CO_2_/H_2_ separation selectivity) at 105 °C.

**Figure 8:**
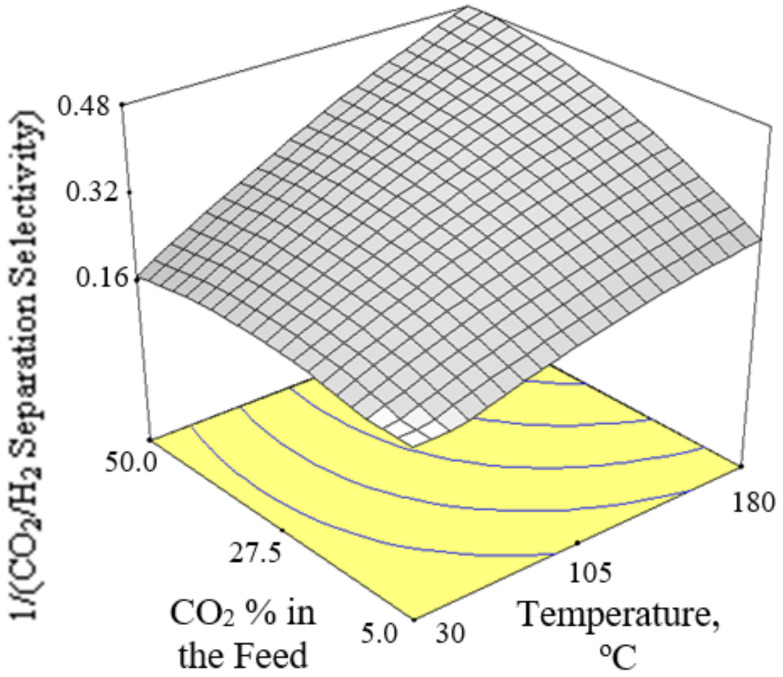
Effect of interaction between CO_2_ % in the feed and temperature on the 1/(CO_2_/H_2_ separation selectivity) at 300 kPa pressure difference.

Figure 6, Figure 7 and Figure 8 present the plots showing the effect of interaction between different independent variables on the 1/(CO_2_/H_2_ separation selectivity). It is shown in Figure 6 that the 1/(CO_2_/H_2_ separation selectivity) increased when the temperature increased from 30 to 180 °C. CO_2_ adsorbed more strongly on Ba-SAPO-34 membrane than H_2_, and hence permeated faster through the membrane pore despite its larger molecule kinetic diameter than H_2_ [57]. Therefore, the CO_2_/H_2_ separation selectivities obtained in the current study were more than 1. The values became less than 1 when the CO_2_/H_2_ separation selectivities were inversed. At temperature as low as 30 °C, strong CO_2_ adsorption on the membrane inhibited the adsorption and permeance of H_2_. The degree of CO_2_ inhibition toward H_2_ adsorption reduced due to lower CO_2_ surface coverage when the temperature increased [39]. The CO_2_ permeance decreased but the H_2_ permeance increased, led to a drop in CO_2_/H_2_ separation selectivity or in other words, an increase in 1/(CO_2_/H_2_ separation selectivity) when the temperature increased. When the CO_2_ % in the feed increased, the 1/(CO_2_/H_2_ separation selectivity) increased, as can be seen in Figure 7 and Figure 8.

### 3.4. Optimization Studies

The goal set for the responses and variables, that need to be satisfied simultaneously for optimizing the responses by using Design Expert, is presented in Table 5. It was the goal for the responses to minimize the 1/(CO_2_/H_2_ separation selectivity) and the 1(CO_2_ permeance), or in other words, to maximize the CO_2_/H_2_ separation selectivity and the CO_2_ permeance.

Design Expert generated solutions (optimum conditions) with different total desirability as shown in Table 6. The desirability function approach was applied in the RSM to optimize the operating conditions in the present work. 

This approach was applied to determine the operating condition that results in response to the highest desirability. Each estimated response variable was transformed into an individual desirability value, *d_i_*, using the desirability function [58]. The value of the desirability varies over the range

(8) $$0\leq d_{i}\leq 1$$

where (*d_i_* = 1) reflects a completely ideal response value and (*d_i_* = 0) reflects a completely undesirable response value. Then, the individual desirability values were combined in order to determine the value of the total desirability, *D*, as shown in Equation (9).

(9) D=d1×d2×…×dm1m

where *m* is the number of responses. In view of the highest total desirability of 0.996 displayed by Solution 1 as shown in Table 6. Solution 1 was selected as the optimum condition. With the optimum condition of Solution 1, 1/(CO_2_/H_2_ separation selectivity) value of 0.086 and the 1/(CO_2_ permeance) value of 0.052 (×10^−7^ mol/m^2^ s Pa)^−1^, were attained, which are equivalent to CO_2_/H_2_ separation selectivity of 11.6 and CO_2_ permeance of 19.23 × 10^−7^ mol/m^2^ s Pa.

An additional five experiments were conducted at the optimum operating condition (temperature of 30 °C, pressure difference of 100 kPa, 5% CO_2_ in the feed) generated by Design of Experiments in order to check the accuracy of the Design of Experiments. The separation result is presented in Table 7. The experimental values of CO_2_/H_2_ separation selectivity and CO_2_ permeance were compared with the values predicted by using the models. The attainment of mean error of 3.64% for CO_2_/H_2_ separation selectivity and the mean error of 1.46% for CO_2_ permeance reflects good agreement between the predicted values and the experimental values. This implies that Design of Experiments with RSM is an accurate tool used to model and predict CO_2_/H_2_ separation performance of the membrane in the current work. 

### 3.5. Comparison of CO_2_/H_2_ Separation Performance with the Other Zeolite Membranes Reported in the Literature

The Ba-SAPO-34 zeolite membrane in the present study was also compared for its CO_2_/H_2_ gas separation performance with the other zeolite membranes reported in the literature, and the comparison is shown in Table 8. Yin et al. [59] prepared a stainless-steel-net-supported P/NaX composite, which displayed CO_2_/H_2_ selectivity of ~4.1–6.4 and CO_2_ permeance of ~0.18–1.68 × 10^−7^ mol/m^2^ s Pa. Mirfendereski et al. [60] reported H_2_/CO_2_ selectivity of 0.11–0.22, which is equivalent to CO_2_/H_2_ selectivity of ~9.1–4.5 for ZSM-5 zeolite membranes. In the other studies reported by Aydani et al. [61], the SSZ-13 membrane was synthesized by dynamic rub coating. CO_2_ permeance of 5.8 × 10^−7^ mol/m^2^ s Pa and CO_2_/H_2_ selectivity of 17 were obtained for the synthesized SSZ-13 membrane [61]. On the other hand, CO_2_/H_2_ selectivity of 17 was reported for the DDR membrane by Zito et al. [62].

Xu et al. [63] prepared Na-LTA and Cs-LTA membranes. Xu et al. [63] reported an H_2_/CO_2_ separation factor of 5.9 and 8 for Na-LTA and Cs-LTA membranes, respectively. Moreover, Li et al. [64] reported the synthesis of the AlPO_4_-LTA membrane and obtained an H_2_/CO_2_ separation factor of 7.3 for the AlPO_4_-LTA membrane. The reported H_2_/CO_2_ separation factor values of greater than one for these membranes indicates that these membranes are H_2_-selective. 

## 4. Conclusions

In this study, the CO_2_/H_2_ separation process over Ba-SAPO-34 zeolite membrane was investigated. Modeling and optimization for the responses (CO_2_/H_2_ separation selectivity and CO_2_ permeance) as a function of the independent process variables (CO_2_ % in the feed, pressure difference and temperature) was performed by applying response surface methodology and central composite design, which is available in Design Expert software. The obtained model equations were able to predict the responses over the ranges of CO_2_ % in the feed, pressure difference and temperature studied. In addition, optimum CO_2_ permeance of 19.23 × 10^−7^ mol/m^2^ s Pa and CO_2_/H_2_ separation selectivity of 11.6 were obtained at 5% CO_2_ in the feed, pressure difference of 100 kPa and temperature of 30 °C for the Ba-SAPO-34 zeolite membrane.

## Figures and Tables

**Table 1:** Independent variables with ranges for CO_2_/H_2_ separation studies in the current study.

Variable (Unit)	Level and Range		
	−1	0	+1
CO_2_ % in the feed (%)	5.0	27.5	50.0
Pressure difference (kPa)	100	300	500
Temperature (°C)	30	105	180

**Table 2:** Independent variables and responses for the CO_2_/H_2_ separation studies.

Run	Variable			Response	
	A	B	C	CO_2_ Permeance (×10^−7^ mol/m^2^ s Pa)	CO_2_/H_2_ Separation Selectivity
	Temperature (°C)	Pressure Difference (kPa)	CO_2_ % in the Feed		
1	30	100	5	19.23	12.2
2	180	100	5	6.15	3.1
3	30	500	5	6.08	5.1
4	180	500	5	6.51	4.0
5	30	100	50	3.85	6.6
6	180	100	50	2.11	1.8
7	30	500	50	2.39	5.0
8	180	500	50	1.96	2.3
9	30	300	27.5	3.74	5.3
10	180	300	27.5	2.71	2.4
11	105	100	27.5	4.17	3.0
12	105	500	27.5	3.21	3.1
13	105	300	5	6.98	4.4
14	105	300	50	2.36	3.0
Repeated Runs					
15	105	300	27.5	3.32	3.0
16	105	300	27.5	3.30	3.1
17	105	300	27.5	3.31	3.1
18	105	300	27.5	3.33	3.1
19	105	300	27.5	3.31	3.0
20	105	300	27.5	3.31	3.0
Mean				3.31	3.05
Standard Deviation				0.01	0.05

**Table 3:** ANOVA of the CO_2_ permeance.

Source	Sum of Squares	Degree of Freedom	Mean Square	F Value	Prob > F
Model	0.260	9	0.029	29,876.61	<0.0001
A	0.026	1	0.026	26,945.27	<0.0001
B	0.014	1	0.014	14,341.69	<0.0001
C	0.200	1	0.200	20.81 × 10^−6^	<0.0001
A^2^	7.589 × 10^−4^	1	7.589 × 10^−4^	794.40	<0.0001
B^2^	1.847 × 10^−3^	1	1.847 × 10^−3^	1932.97	<0.0001
C^2^	8.992 × 10^−4^	1	8.992 × 10^−4^	941.26	<0.0001
AB	7.434 × 10^−3^	1	7.434 × 10^−3^	7781.67	<0.0001
AC	5.317 × 10^−3^	1	5.317 × 10^−3^	5566.44	<0.0001
BC	1.046 × 10^−3^	1	1.046 × 10^−3^	1094.58	<0.0001
Residual	9.553 × 10^−6^	10	9.553 × 10^−7^	-	-
Lack of Fit	5.140 × 10^−6^	5	1.028 × 10^−6^	1.16	0.4357
Pure Error	4.413 × 10^−6^	5	8.826 × 10^−7^	-	-
Cor Total	0.260	19	-	-	-

**Table 4:** ANOVA of CO_2_/H_2_ separation selectivity.

Source	Sum of Squares	Degree of Freedom	Mean Square	F Value	Prob > F
Model	0.220	9	0.024	391.84	<0.0001
A	0.140	1	0.140	2209.94	<0.0001
B	3.112 × 10^−4^	1	3.112 × 10^−4^	5.03	0.0488
C	0.036	1	0.036	586.84	<0.0001
A^2^	1.156 × 10^−3^	1	1.156 × 10^−3^	18.68	0.0015
B^2^	2.755 × 10^−4^	1	2.755 × 10^−4^	4.45	0.0610
C^2^	4.118 × 10^−3^	1	4.118 × 10^−3^	66.57	<0.0001
AB	0.017	1	0.017	269.22	<0.0001
AC	0.015	1	0.015	243.05	<0.0001
BC	1.851 × 10^−3^	1	1.851 × 10^−3^	29.93	0.0003
Residual	6.186 × 10^−4^	10	6.186 × 10^−5^	-	-
Lack of Fit	5.036 × 10^−4^	5	1.007 × 10^−4^	4.39	0.0655
Pure Error	1.150 × 10^−4^	5	2.300 × 10^−5^	-	-
Cor Total	0.220	19	-	-	-

**Table 5:** Goal set for optimization of the studies of CO_2_/H_2_ separation.

Name		Goal	Lower Limit	Upper Limit
Variable	Temperature, °C	Within range	30	100
	Pressure Difference, kPa	Within range	100	500
	CO_2_ % in the Feed	Within range	5	50
Response	1/(CO_2_ Permeance), (×10^−7^ mol/m^2^ s Pa)^−1^	Minimum	0.05	0.51
	1/(CO_2_/H_2_ Separation Selectivity)	Minimum	0.08	0.56

**Table 6:** Optimum conditions for the 1/(CO_2_/H_2_ separation selectivity) and the 1/(CO_2_ permeance) generated by Design Expert.

Solu-tion	Temperature, °C	Pressure Difference, kPa	CO_2_ % in the Feed	1/(CO_2_ Permeance), (×10^−7^ mol/m^2^ s Pa)^−1^	1/(CO_2_/H_2_ Separation Selectivity)	Total Desirability
1	30.00	100.00	5.00	0.052	0.086	0.996
2	30.00	100.00	5.35	0.056	0.087	0.990
3	30.01	107.02	5.00	0.056	0.087	0.990
4	30.07	114.32	5.00	0.060	0.088	0.985
5	30.00	102.66	6.03	0.060	0.091	0.982
6	31.91	100.00	5.93	0.059	0.094	0.980
7	40.46	100.00	5.00	0.056	0.107	0.969

**Table 7:** The results of the additional CO_2_/H_2_ separation experiments conducted at optimum operating condition generated by Design of Experiments.

Run	CO_2_ Permeance (×10^−7^ mol/m^2^ s Pa)		ΔError (%)	CO_2_/H_2_ Separation Selectivity		ΔError (%)
	Experimental	Predicted (Design of Experiments)		Experimental	Predicted (Design of Experiments)	
1	19.11	19.23	0.62	12.1	11.6	4.13
2	18.99	19.23	1.25	11.9	11.6	2.52
3	19.01	19.23	1.14	12.2	11.6	4.92
4	19.52	19.23	1.51	12.2	11.6	4.92
5	18.70	19.23	2.76	11.8	11.6	1.69
Mean Error			1.46			3.64
Standard Deviation			0.71			1.31

**Table 8:** Comparison of CO_2_/H_2_ separation performance with the other zeolite membranes reported in the literature.

Zeolite Membrane	CO_2_/H_2_ Selectivity * or H_2_/CO_2_ Separation Factor ^+^	Reference
Ba-SAPO-34	1.8–12.2 *	Present study
P/NaX	~4.1–6.4 *	[59]
ZSM-5	~9.1–4.5 *	[60]
SSZ-13	17 *	[61]
DDR	17 *	[62]
Na-LTA	5.9 ^+^	[63]
Cs-LTA	8 ^+^	[63]
AlPO_4_-LTA	7.3 ^+^	[64]

* CO_2_/H_2_ Selectivity; ^+^ H_2_/CO_2_ Separation Factor.

## Data Availability

Not applicable.
